# Right-sided Bochdalek hernia in an adult with hepatic malformation and intestinal malrotation

**DOI:** 10.1186/s40792-021-01232-5

**Published:** 2021-07-17

**Authors:** Naoki Enomoto, Kazuhiko Yamada, Daiki Kato, Shusuke Yagi, Hitomi Wake, Kyoko Nohara, Nobuyuki Takemura, Tomomichi Kiyomatsu, Norihiro Kokudo

**Affiliations:** Department of Surgery, Center Hospital of the National Center for Global Health and Medicine, 1-21-1 Toyama, Shinjuku-ku, Tokyo, 162-8655 Japan

**Keywords:** Bochdalek hernia, Adult, Right-sided, Hepatic malformation, Intestinal malrotation, Three-dimensional simulation

## Abstract

**Background:**

Bochdalek hernia is a common congenital diaphragmatic defect that usually manifests with cardiopulmonary insufficiency in neonates. It is very rare in adults, and symptomatic cases are mostly left-sided. Diaphragmatic defects generally warrant immediate surgical intervention to reduce the risk of incarceration or strangulation of the displaced viscera.

**Case presentation:**

A 47-year-old woman presented with dyspnea on exertion. Computed tomography revealed that a large part of the intestinal loop with superior mesenteric vessels and the right kidney were displaced into the right thoracic cavity. Preoperative three-dimensional (3D) simulation software visualized detailed anatomy of displaced viscera and the precise location and size of the diaphragmatic defect. She underwent elective surgery after concomitant pulmonary hypertension was stabilized preoperatively. The laparotomic approach was adopted. Malformation of the liver and the presence of intestinal malrotation were confirmed during the operation. The distal part of the duodenum, jejunum, ileum, colon, and right kidney were reduced into the abdominal cavity consecutively. A large-sized oval defect was closed with monofilament polypropylene mesh. No complications occurred postoperatively.

**Conclusion:**

Symptomatic right-sided Bochdalek hernia in adults is exceedingly rare and is frequently accompanied by various visceral anomalies. Accurate diagnosis and appropriate surgical repair are crucial to prevent possible incarceration or strangulation. The preoperative 3D simulation provided comprehensive information on anatomy and concomitant anomalies and helped surgeons plan the operation meticulously and perform procedures safely.

## Introduction

Bochdalek hernia is a diaphragmatic hernia located on the posterolateral portion of the diaphragm [1]. Most cases are left-sided and are commonly diagnosed during the neonatal period. Neonatal Bochdalek hernia frequently compromises the cardiopulmonary function and carries a high rate of mortality.

Right-sided Bochdalek hernia is extremely rare in adults. Early closure of the right pleuroperitoneal canal and the buttressing effect of the liver may explain the reason [2]. We present an adult case of right-sided Bochdalek hernia accompanied by malformation of the liver and intestinal malrotation.

## Case presentation

The patient was a 47-year-old woman who presented with dyspnea on exertion. Her medical history was remarkable for asthma and ovarian cyst. At regular health check-ups during her elementary school years, chest radiographs appeared abnormal; however, she never had any opportunities for further examinations until this presentation.

**Fig. 1:**
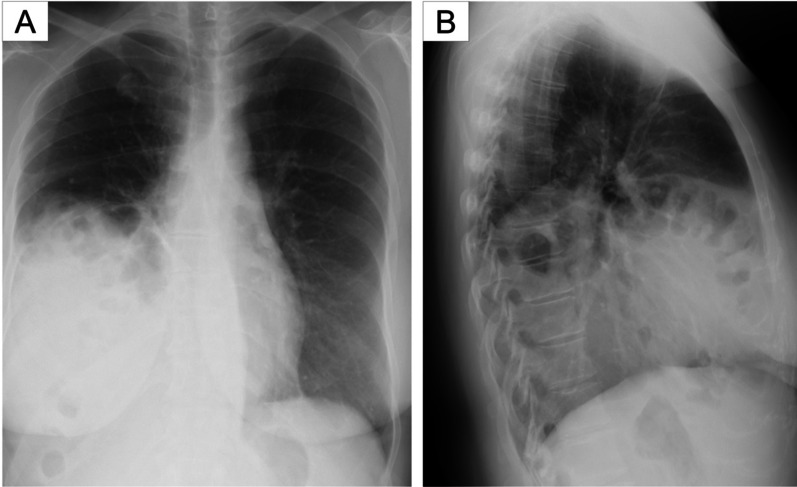
Preoperative chest radiography. **a** Frontal chest radiography showed a massive area of opacity in the right lung field. **b** Lateral chest radiography showed intestinal gas in opacity in the lung fields

**Fig. 2:**
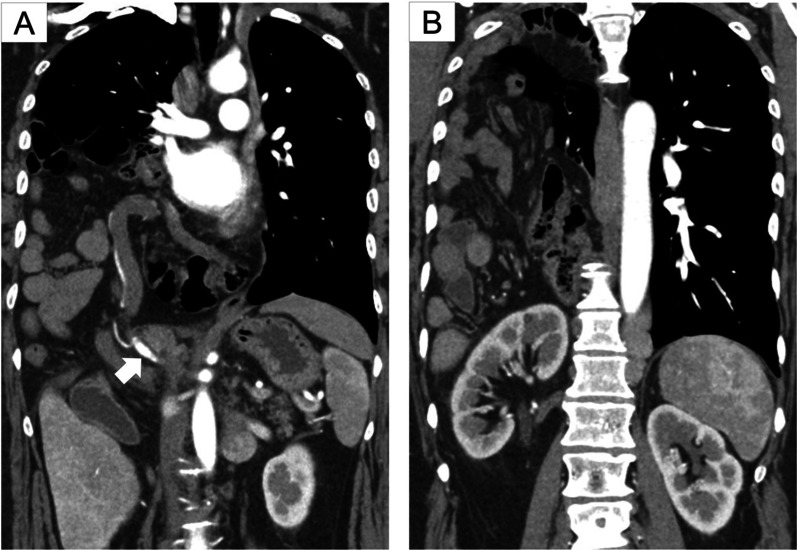
Coronal views of preoperative contrast medium-enhanced thoracoabdominal computed tomographic scans. **a** Small intestine, the colon, and the main trunk of the superior mesenteric artery (white arrow) had herniated through the diaphragmatic defect into the right thoracic cavity. **b** Right kidney was detached from the retroperitoneum and had also herniated into the right thoracic cavity

Chest radiographs showed a massive area of opacity mixed with intestinal gas in the right lung field (Fig. 1). Computed tomography (CT) scans revealed that both the right kidney and the portion of the alimentary tract from the distal part of the duodenum to the transverse colon had herniated into the right thoracic cavity (Fig. 2). A cardiac catheterization study demonstrated elevated pulmonary capillary wedge pressure (31 mm Hg) and elevated pulmonary arterial pressure (53 mm Hg). Right-sided Bochdalek hernia and pulmonary hypertension were diagnosed. After her cardiopulmonary function was stabilized with medical therapy, she was referred to the surgical department for operative repair of the diaphragmatic hernia.

**Fig. 3:**
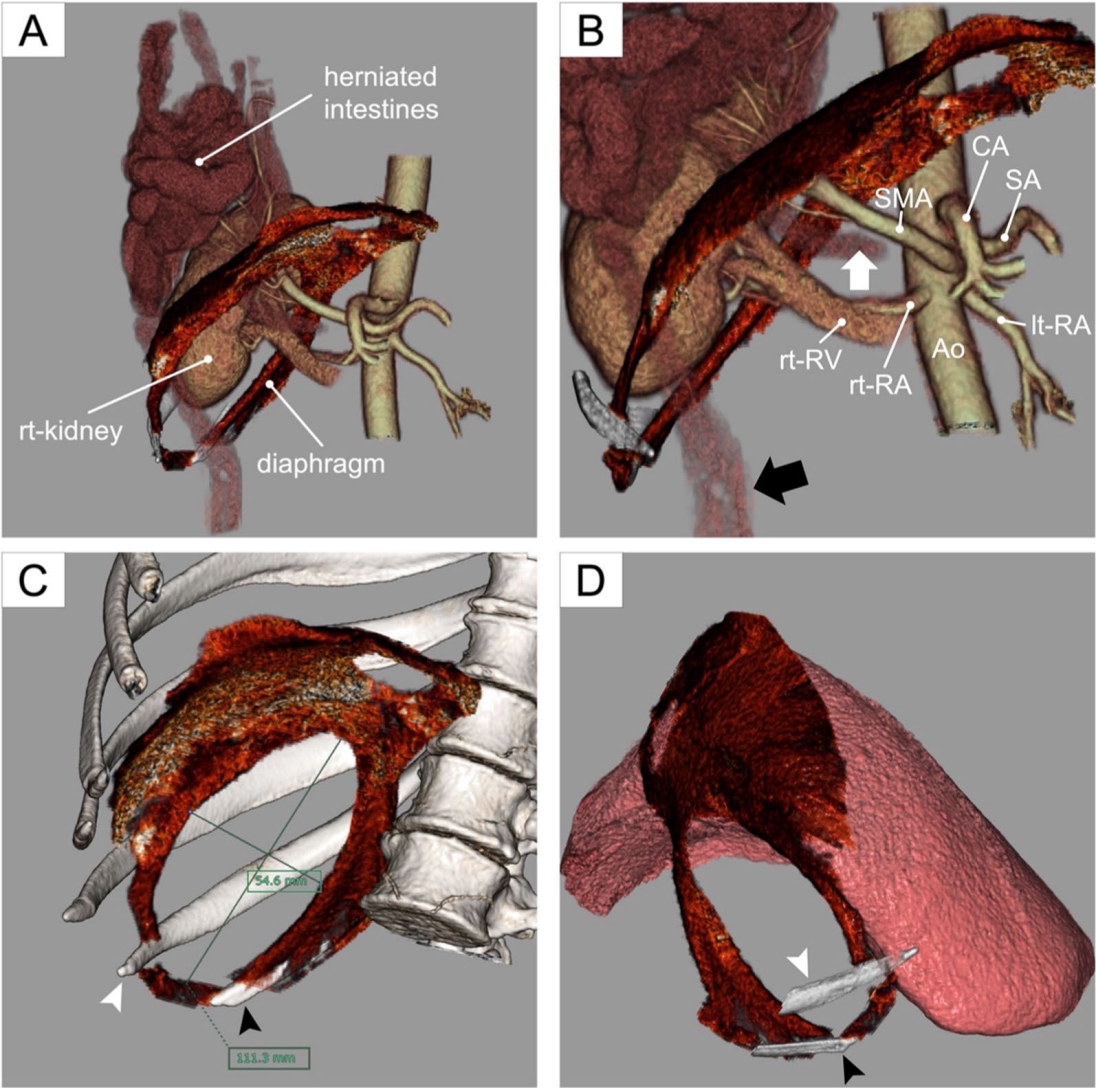
Preoperative three-dimensional (3D) simulation. **a** Herniated intestines and the right kidney occupied the right thoracic cavity. **b** Portion of the alimentary tract [from the distal part of duodenum (white arrow) to the proximal part of transverse colon (black arrow)] and the main trunk of superior mesenteric artery had herniated through the diaphragmatic defect. **c** Oval diaphragmatic defect was present on the posterolateral portion of the diaphragm. 3D simulation precisely visualized the location and size of the defect. The eleventh rib (white arrowhead) and the twelfth rib (black arrowhead) with intercostal muscles constituted the posterolateral part of the defect. **d** Right lobe of the liver was flattened, thus leaving the large diaphragmatic defect uncovered. *Ao* aorta, *CA* celiac artery, *lt-RA* left renal artery, *rt-kidney* right kidney, *rt-RA* right renal artery, *rt-RV* right renal vein, *SA* splenic artery, *SMA* superior mesenteric artery

To visualize the anatomy and vasculature preoperatively, we used 3D analysis software (Synapse Vincent; Fujifilm, Tokyo, Japan) to generate three-dimensional (3D) images from thin-section reconstructed CT data (Fig. 3). These images showed that the superior mesenteric artery (SMA) and vein (SMV) had herniated through the diaphragmatic defect. The eleventh and twelfth ribs with intercostal muscle constituted the posterolateral part of the defect. The size of the elliptical defect was estimated to be 11 $\times$ 5 cm. The liver was flattened in shape, which left the large diaphragmatic defect uncovered. The right kidney was detached from the retroperitoneum and had also herniated through the defect.

A transabdominal approach was selected given the difficulty in reducing the huge volume of herniated contents through the transthoracic approach. We adopted a laparotomic approach, because reducing the main trunks of SMA and SMV laparoscopically might have been unsafe, and any resulting pneumoperitoneum might have worsened the preexisting cardiopulmonary burden.

**Fig. 4:**
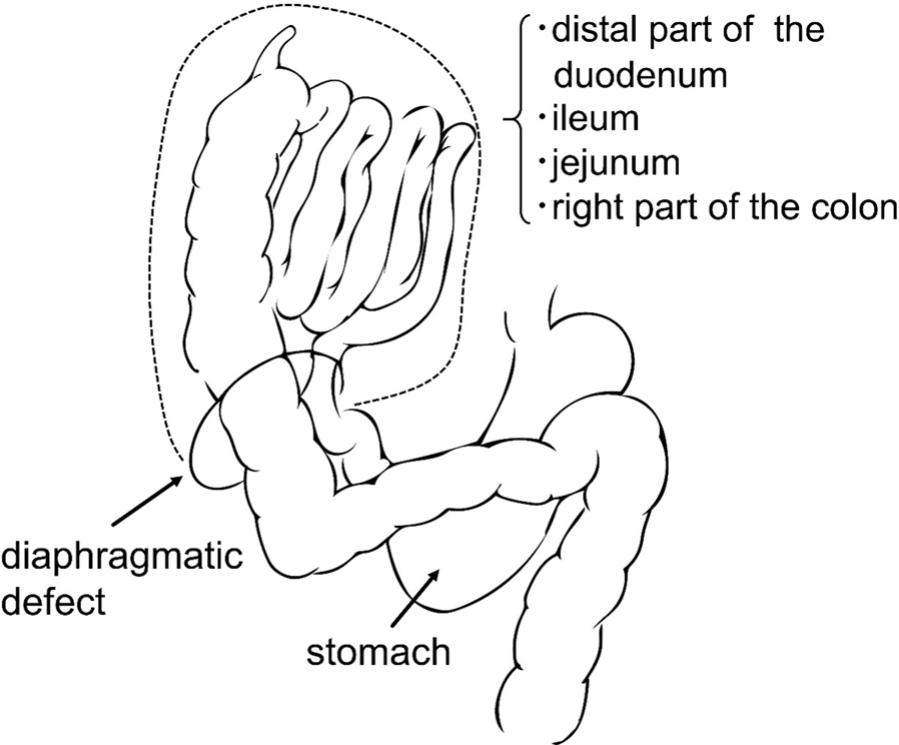
Schematic illustration of the herniated digestive tract and intestinal malrotation. The distal part of the duodenum, ileum, jejunum, and the right part of the colon were herniated into the right thoracic cavity. The duodenum, cecum, and the ascending colon had lost the fixation to the retroperitoneum

The patient was placed supine while under general anesthesia, and a J-shaped incision was made in the abdomen. The flattened appearance of the liver was confirmed. We fully mobilized the right lobe of the liver to expose the diaphragm. The small intestine and ascending colon were attached to the common mesentery without any retroperitoneal fixation. We confirmed intestinal malrotation (IM) and the absence of the ligament of Treitz (Fig. 4). The IM was classified as a nonrotation pattern.

**Fig. 5:**
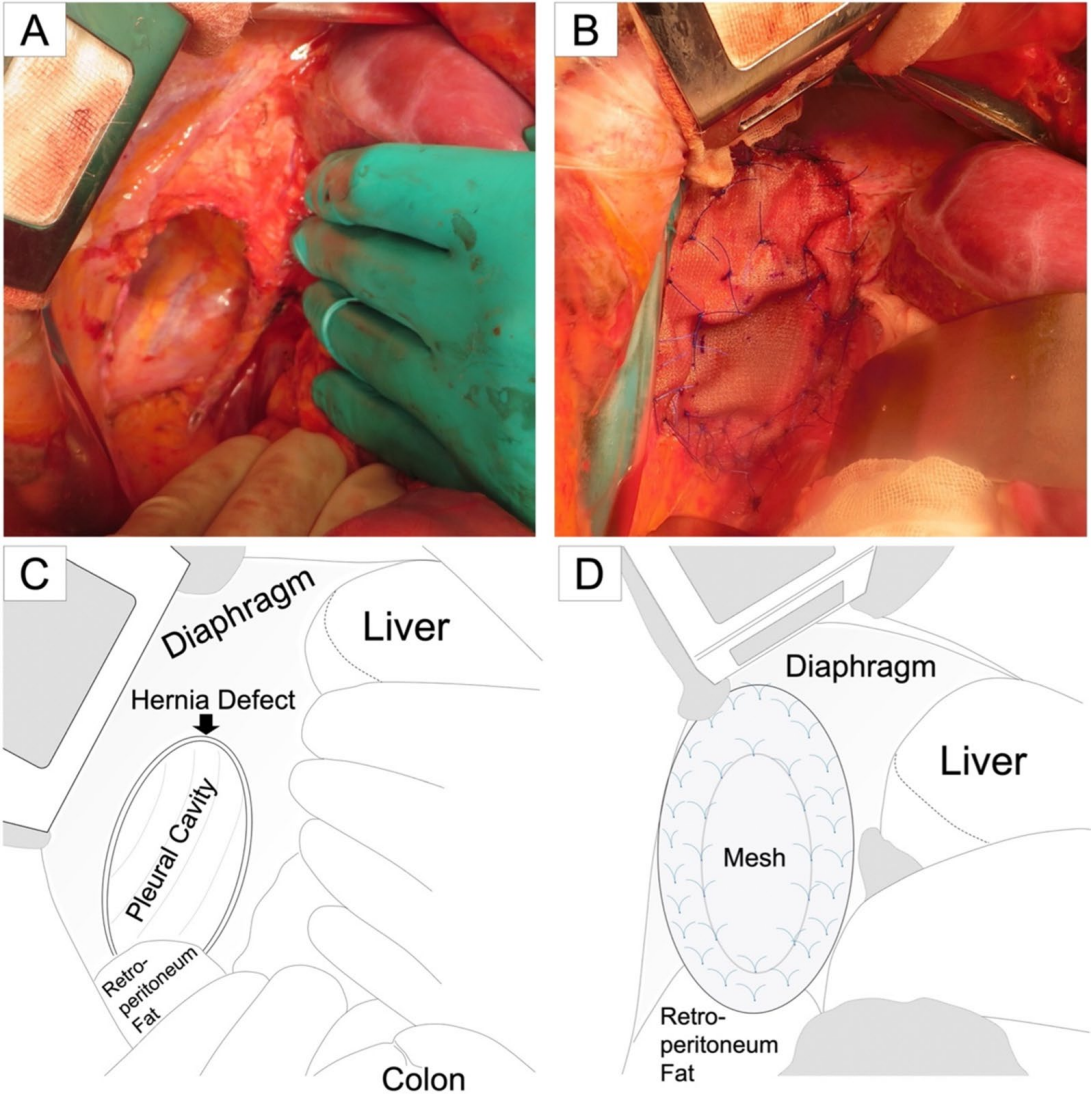
Intraoperative photographs. **a** Distal part of the duodenum, ileum, jejunum, the right part of the colon, and the right kidney were reduced into the abdominal cavity consecutively. The defect was exposed on the posterolateral portion of the diaphragm, as predicted. **b** Large oval defect was closed with monofilament polypropylene mesh. **c** Schematic illustration of the photograph A. **d** Schematic illustration of the photograph B

We gently dissected the adhesion between the intestines and the rim of the defect. First, the colon was pulled back to the abdominal cavity, and then the distal part of the duodenum, ileum, and jejunum were sequentially reduced. There was neither adhesion nor hernia sac in the thoracic cavity. We meticulously manipulated the main trunks of SMA and SMV during their reduction. The defect was completely exposed following the reduction of the right kidney. The diameter of the defect was indeed 11 $\times$ 5 cm, as estimated. Monofilament polypropylene mesh (Ventralight™ ST mesh; Becton, Dickinson and Company, Franklin Lakes, NJ, USA) was cut in an oval with sufficient margins and fixed to the diaphragm circumferentially (Fig. 5). Intestinal repositioning or fixation was not performed, because nonrotation pattern IM has a low risk of intestinal volvulus.

**Fig. 6:**
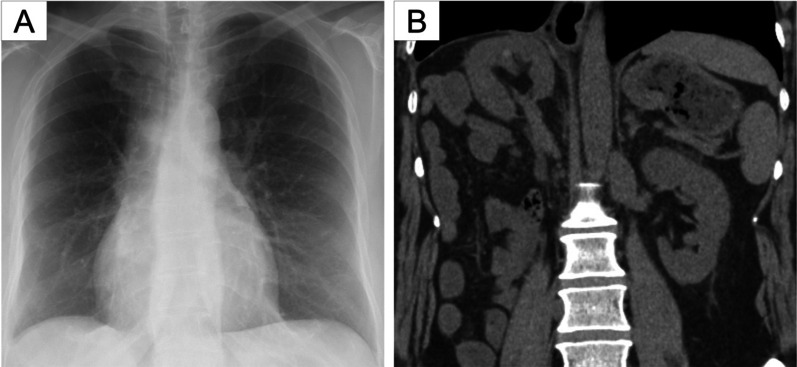
Postoperative chest radiograph (**a**) and coronal abdominal computed tomographic (CT) scan (**b**). **a** Massive area of opacity had completely disappeared. **b** No recurrence of the diaphragmatic hernia was confirmed 20 months after the operation

The patient was discharged without any complications on postoperative day 10. Dyspnea on exertion was relieved, and the severity of pulmonary hypertension gradually lessened over time. The pulmonary capillary wedge pressure and mean pulmonary arterial pressure decreased to 10 and 20 mm Hg, respectively. She exhibited no recurrence of the hernia at the 20-month follow-up visit (Fig. 6).

## Discussion

By the eighth week of embryonic development, the pleuroperitoneal membranes have fused with the septum transversum centrally, the mesoesophagus mediodorsally, and the musculature of the lateral body wall [1, 3]. Failure to fuse leads to several types of diaphragmatic hernia. Failure of the septum transversum to fuse anteriorly leads to Morgagni hernias. Posterolateral defects (Bochdalek hernias) were first described by Victor Alexander Bochdalek in 1848. Bochdalek hernia occurs as the consequence of the incomplete fusion of the pleuroperitoneal membrane, the dorsal esophageal mesentery, and the body wall [4].

Most cases of Bochdalek hernia with large defects are diagnosed during the neonatal period due to the accompanying severe respiratory failure. Congenital Bochdalek hernia is the most common congenital diaphragmatic hernia (CDH), accounting for 90% to 95% of such hernias [5, 6]. The incidence of congenital Bochdalek hernia has been estimated to be 1 per 2000 to 12,500 live births, and 85% of congenital Bochdalek hernias are left-sided [2].

Bochdalek hernia is rarely found in adults. It could occur after trauma, pregnancy, abdominal surgery, and other events that increase intra-abdominal pressure and exacerbate subtle diaphragmatic defects that are too small to cause symptoms early in life. Asymptomatic adult Bochdalek hernia is usually found incidentally with imaging modalities, such as chest X-ray or multidetector-row CT. In a review of 13,138 abdominal CT scans performed to rule out metastatic disease in patients with known malignancy, the incidence of asymptomatic adult Bochdalek hernia was 0.17%; the defect was right-sided in 68% of the affected patients, and 77% of the affected patients were female [7]. Of note, asymptomatic cases of adult Bochdalek hernia were found more on the right side than on the left side, whereas congenital Bochdalek hernia is generally found on the left side. However, of 109 adult patients with symptomatic Bochdalek hernia who underwent surgical repair, 82% had left-sided defects [8]. A buttressing effect of the liver that prevents the displacement of abdominal viscera into the thoracic cavity may explain the difference in laterality between asymptomatic and symptomatic Bochdalek hernia in adults. Right-sided Bochdalek hernia in adults is extremely rare. Through an extensive search with the Japan Medical Abstracts Society and PubMed, we identified and analyzed 43 case reports concerning right-sided Bochdalek hernia in adults. Cases without surgical repair and operative details and the case reports without Japanese or English translations were excluded. These are summarized in Table 1. The median age was 64 years, and 22 patients (65%) were female. The median size of the diaphragmatic defect was 7 cm in diameter. Twenty patients (57%) presented with abdominal or gastrointestinal symptoms, while 17 cases (49%) had chest or respiratory symptoms. As to surgical routes, 23 cases (68%), 3 cases (9%), and 8 cases (23%) had abdominal, thoracic, and combined thoracoabdominal approaches, respectively. Seven patients underwent minimally invasive surgery. Mesh repair, suture repair, and the combined method were selected in 10 cases (29%), 22 cases (65%), and 2 cases (6%), respectively.

**Table.1:** List of previously reported cases with right-sided Bochdalek hernia in adults

Author	Year	Age	Sex	Chief complain	Anomaly	Herniated viscera	Size of defect	Route	Approach	Sac	Repair
Sato	1973	48	M	Chest pain, dyspnea	Visceral inversion Mesenterium commune	Jejunum, ileum	6 × 3 cm	A	Laparotomic	Unknown	Suture
Ochi	1984	43	M	Abdominal pain, abdominal distension	Gastric volvulus	Duodenum, liver, gallbladder	Chicken egg size	T/A	Thoracotomic Laparotomic	Absent	Suture
Zaima	1984	47	F	Chest pain	Mesenterium commune Thinning of the right hepatic lobe	Small intestine, colon	6 × 4 cm	A	Laparotomic	Absent	Suture
Sinha	1989	70	F	Chest pain, shortness of breath	None	Colon	3 × 3 cm	A	Laparotomic	Unknown	Suture
Idota	1997	68	F	Abdominal pain	Thinning of the liver Incomplete fixation of the colon	Ileum, colon	8 × 5 cm	A	Laparotomic	Absent	Suture
Mar Fan	1999	36	F	Abdominal pain	None	Small intestine	5 cm	A	Laparotomic	Absent	Suture
Zenda	2000	69	M	Abdominal pain	Thinning of the liver	Ileum, colon, gallbladder	15 × 10 cm	A	Laparotomic	Unknown	Suture
Kanazawa	2002	63	F	Abdominal pain, dyspnea	Thinning of the liver	Colon, kidney	12 cm	T/A	Thoracotomic Laparotomic	Unknown	Suture
Kiriyama	2002	41	F	Cough, sputum	Thinning of the liver Intestinal malrotation	Stomach, duodenum, small intestine	10 × 7 cm	A	Laparotomic	Absent	Mesh
Masuda	2007	80	F	Abdominal pain, vomiting	Incomplete fixation of the duodenum	Stomach, duodenum	Unknown	A	Laparotomic	Unknown	Suture
Rosen	2007	50	M	Dyspnea	None	Retroperitoneal fat	2 × 2 cm	A	Laparoscopic	Unknown	Mesh
Hirose	2008	64	M	Cough, hemoptysis	None	Colon, liver, kidney	8 × 8 cm	T	Thoracotomic	Absent	Mesh
Fraser	2009	75	F	Cough	None	Small intestine, colon, kidney	8 × 5 cm	T/A	Thoracoscopic Laparoscopic	Present	Mesh
Laakesonen	2009	38	F	Abdominal pain	None	Colon, liver	10 cm	T/A	Thoracotomic Laparoscopic	Absent	Suture + mesh
Matsushita	2009	21	F	No complaint	None	Small part of the liver	3 cm	T/A	Thoracotomic Laparotomic	Present	Suture
Murakami	2010	85	M	Abdominal pain	Thinning of the liver	Ileum	3 cm	A	Laparotomic	Absent	Mesh
Nishiwaki	2011	59	M	Dyspnea	None	Small intestine, liver	10 × 8 cm	T	Thoracotomic	Absent	Mesh
Choi	2012	29	M	Chest pain, dyspnea	Thinning of the liver	Duodenum, ileum, jejunum, colon, pancreas head	7 cm	T/A	Thoracotomic Laparotomic	Unknown	Mesh
Almeida	2013	49	F	Cough, sputum	None	Colon	6 × 3 cm	A	Laparotomic	Absent	Suture
Miyake	2013	80	M	Abdominal pain	Unknown	Small intestine	Unknown	A	Laparotomic	Unknown	Suture
Mizoguchi	2013	71	F	Dyspnea	Mesenterium commune	Small intestine, colon	4 × 3.5 cm	A	Laparotomic	Absent	Mesh
Patle	2013	50	F	Dyspnea, abdominal discomfort	Incomplete fixation of the liver	Colon, kidney	10 × 8 cm	A	Laparoscopic	Absent	Suture + mesh
Debergh	2014	38	F	Abdominal pain	None	Small intestine	5 × 4 cm	A	Laparoscopic	Absent	Suture
Salústio	2014	50	F	Abdominal pain, vomiting	Intestinal malrotation Incomplete fixation of the colon and the liver	Small intestine	Unknown	A	Laparotomic	Unknown	Suture
Watanabe	2015	65	F	Abdominal pain, dyspnea	None	Colon, small part of the liver	5 cm	A	Laparotomic	Absent	Suture
Kohli	2016	22	M	Chest pain, shortness of breath	Abnormal rotation of the liver	Small intestine, colon, gallbladder	10 cm	T/A	Thoracotomic Laparoscopic	Present	Mesh
Ohtsuka	2016	89	F	Abdominal pain, vomiting, dyspnea	Incomplete fixation of the colon	Ileum, colon	4.5 × 3 cm	A	Laparotomic	Absent	Suture
Moro	2017	89	F	Abdominal pain	None	Ileum	3 × 2 cm	A	Laparotomic	Unknown	Suture
Jambhekar	2018	74	M	Nausea, vomiting	None	Colon, liver	8 × 6 cm	A	Robotic	Unknown	Suture
Hojo	2019	70	F	Abdominal pain	Thinning of the liver incomplete fixation of the colon	Ileum, colon, liver	6 × 5 cm	A	Laparoscopic	Absent	Suture
Toda	2019	72	M	Nausea	None	Retroperitoneal fat	8 × 5 cm	T	Thoracotomic	Absent	Suture
Onishi	2020	66	F	Abdominal pain	Thinning of the liver	Colon, liver, gallbladder	7 × 5 cm	T/A	Thoracoscopic Laparoscopic	Absent	Suture
Kyoku	2021	77	F	Abdominal pain	None	Small intestine	3 × 3 cm	A	Laparotomic	Absent	Suture
Present case	2021	47	F	Dyspnea	Thinning of the liver Intestinal malrotation	Duodenum, small intestine, colon, kidney	11 × 5 cm	A	Laparotomic	Absent	Mesh

*A* Abdominal, *F* Female, *M* Male, *T* Thoracic, *T/A* Thoracoabdominal

Bochdalek hernia is known to accompany several types of congenital anomalies, such as mesenterium commune, situs inversus, intestinal malrotation (IM), gastric volvulus, and malformation of the liver [9]. IM is a congenital anomaly that results from an abnormal rotation of the midgut. It occurs as the midgut returns to the abdominal cavity during the ninth to tenth week of gestation. The incidence of symptomatic IM is estimated to be 1 in 500 live births [10]. IM is classified into four patterns; normal, incomplete, reverse, and nonrotation based on the presumed timing of the developmental failure [11]. The patient had a nonrotation pattern of IM in this presentation. The nonrotation pattern is the most frequent in adults and is caused by the failure to complete the final rotation of 180° following the normal first 90° rotation [12]. Nonrotation pattern IM has a low risk of volvulus; thus intestinal repositioning is not necessary in cases without intestinal obstruction [13].

Hypoplasia or atrophy of the right lobe of the liver can facilitate herniation of abdominal organs through the uncovered defect and is frequently observed in right-sided Bochdalek hernia. Malformation of the right lobe of the liver may be the consequence of compromised portal perfusion owing to the long-standing compression effect of displaced organs.

Common symptoms of Bochdalek hernia in adults include dyspnea, cough, chest pain, abdominal pain, and vomiting [2]. Bochdalek hernia in adults is often difficult to diagnose, because the symptoms are often mild and nonspecific [3, 13]. The use of appropriate imaging modalities, such as magnetic resonance imaging or CT, is crucial for accurate diagnosis. If diagnosis and treatment are delayed, incarceration or strangulation of herniated organs can occur [14, 15]. Surgical treatment is recommended regardless of symptoms or laterality, in view of the potential risk of visceral complications [16, 17].

Bochdalek hernia is surgically repaired with a thoracic, abdominal, or combined thoracoabdominal approach [8]. The advantages of the thoracic approach over the abdominal approach are direct visualization of herniated contents and nonnegative intrapleural pressure, which facilitates the reduction [18]. Through the thoracic approach, adhesions between the displaced viscera and lungs or pleurae can be safely dissected, although few intrapleural adhesions were found in previous reports. In contrast, the advantage of the abdominal approach is easier recognition and management of possible intestinal strangulation and concomitant anomalies of abdominal organs [19]. The abdominal approach is more popular than the thoracic approach and might be suitable in cases complicated by potential strangulation, ischemia, or visceral anomalies. Minimally invasive surgeries with thoracoscopic, laparoscopic, and robotic procedures are increasingly used for Bochdalek hernia on both sides recently [19–23].

The diaphragmatic defect is usually closed with sutures for minor defects. The use of mesh might be recommended for cases with large defects or fragile diaphragm texture, or patients who are extremely obese. We selected mesh repair, considering the large size of the defect and the obese body shape in the present case.

Giant abdominal hernia is associated with postoperative abdominal compartment syndrome (ACS) following the reduction of herniated contents. The use of appropriate mesh, resection of intestines to lessen the volume of the abdominal organs, and delayed abdominal closure are measures to decrease the risk of ACS. ACS after repair of CDH in neonates is frequently reported [24]. Delayed abdominal closure with the use of silicone silo bags or vacuum-assisted closure devices is performed to prevent ACS in neonates with a large volume of herniated content and limited abdominal capacity. In contrast, ACS after repair of diaphragmatic hernia in adults is extremely rare though few cases were reported. The use of sufficiently large-sized mesh, the adequate volume of the abdominal cavity, and the absence of excessive wound tension might contribute to the uneventful course postoperatively in this case.

CDH is associated with respiratory distress and pulmonary hypertension because of pulmonary hypoplasia in neonates [25]. Persistent pulmonary hypertension (PPH) after successful repair of CDH is one of the significant risk factors for morbidity and mortality in infants [26]. The patient had the potential to develop PPH following the surgical repair of Bochdalek hernia, because she had preoperative pulmonary hypertension in the present case. Fortunately, pulmonary capillary wedge pressure and pulmonary arterial pressure gradually decreased within acceptable range after surgery over time.

3D simulation software has been developed to depict anatomical structures and has assisted surgeons in performing precise and safe procedures since the late 1990s [27]. Virtual reality or augmented reality modalities based on 3D data navigate a broad variety of procedures [28–32]. Few prior studies have demonstrated the usefulness of 3D simulation for diaphragmatic hernia surgery. ﻿In this case, the visualization of the displaced viscera and vessels, the deformed liver, and the diaphragmatic defect allowed us to prepare the appropriate mesh and perform safe procedures without any postoperative complications.

## Conclusions

Bochdalek hernia is the most common type of CDH and generally manifests during the neonatal period. Right-sided Bochdalek hernia in adults is extremely rare, and various congenital visceral anomalies accompany most cases. Accurate diagnosis and appropriate surgical repair are crucial for preventing incarceration or strangulation of abdominal organs. Preoperative 3D simulation offers detailed information about the herniated viscera and vessels, the diaphragmatic defect, and concomitant anomalies. We had developed a meticulous operative plan based on the simulation and successfully performed surgical repair safely.

## Data Availability

The data sets analyzed during the current study are not publicly available due to their containing information that could compromise the privacy of research participants but are available from the corresponding author on reasonable request.

## Abbreviations

ACS: Abdominal compartment syndrome
CDH: Congenital diaphragmatic hernia
CT: Computed tomography
IM: Intestinal malrotation
PPH: Persistent pulmonary hypertension
SMA: Superior mesenteric artery
SMV: Superior mesenteric vein
3D: Three-dimensional
